# Supplementary material for: Chromothripsis during telomere crisis is independent of NHEJ, and consistent with a replicative origin
Source: Genome Res. 2019 May;29(5):737–49. doi: 10.1101/gr.240705.118 (PMC6499312; doi:10.1101/gr.240705.118)
Supplement: Supplemental Material [file supp_gr.240705.118_Supplemental_file_1.zip › contigs/annotated_contigs/DB93/contig.2.DB93_length_284_mean_cov_10.161971831.docx]

**DB93_length_284_mean_cov_10.161971831**

```
ACCATTCTTTGGAGTGCCACATTCTGCTGTGGTTTTGCTCCTATAATAGGTCTTTTGCTCTACACCTTTGCAGAGATGCATACAAATGT
 >chr3:114888417-114888586 + E=1e-90
TTCTGCAAAGTACCATAACATCTTAAAGAATGCAGTGTTTGGCCCTGAGGCCATGTGTCTTGAAATTTTTTGTGTGTA|TT|CCCCAGC
                                                                               >chr3:11
TGTGAAATGGGGTGATTAAGGTAGATGTGAGAATTAAATGCAATGATACAGGTACACTTCGCACTGTTCTGAGCACATAGTAAGAGCTC
5434815-115434932 + E=4e-59
GAAAATGTTAACTATAGCT
```
